# Artificial intelligence stenosis diagnosis in coronary CTA: effect on the performance and consistency of readers with less cardiovascular experience

**DOI:** 10.1186/s12880-022-00756-y

**Published:** 2022-02-17

**Authors:** Xianjun Han, Nan Luo, Lixue Xu, Jiaxin Cao, Ning Guo, Yi He, Min Hong, Xibin Jia, Zhenchang Wang, Zhenghan Yang

**Affiliations:** 1 Department of Radiology, Beijing Friendship Hospital, Capital Medical University, No. 95 YongAn Road, Beijing, 100050 People’s Republic of China; 2 Shukun (Beijing) Technology Co., Ltd., Jinhui Bd, Qiyang Rd, Beijing, 100102 People’s Republic of China; 3 Department of Computer Software Engineering, Soonchunhyang University, Asan, South Korea; 4 Beijing University of Technology, Beijing, People’s Republic of China

**Keywords:** Artificial intelligence (AI), CCTA, Coronary artery disease, Coronary stenosis, Inexperience readers

## Abstract

**Background:**

To investigate the influence of artificial intelligence (AI) based on deep learning on the diagnostic performance and consistency of inexperienced cardiovascular radiologists.

**Methods:**

We enrolled 196 patents who had undergone both coronary computed tomography angiography (CCTA) and invasive coronary angiography (ICA) within 6 months. Four readers with less cardiovascular experience (Reader 1–Reader 4) and two cardiovascular radiologists (level II, Reader 5 and Reader 6) evaluated all images for ≥ 50% coronary artery stenosis, with ICA as the gold standard. Reader 3 and Reader 4 interpreted with AI system assistance, and the other four readers interpreted without the AI system. The sensitivity, specificity, positive predictive value (PPV), negative predictive value (NPV) and accuracy (area under the receiver operating characteristic curve (AUC)) of the six readers were calculated at the patient and vessel levels. Additionally, we evaluated the interobserver consistency between Reader 1 and Reader 2, Reader 3 and Reader 4, and Reader 5 and Reader 6.

**Results:**

The AI system had 94% and 78% sensitivity at the patient and vessel levels, respectively, which were higher than that of Reader 5 and Reader 6. AI-assisted Reader 3 and Reader 4 had higher sensitivity (range + 7.2–+ 16.6% and + 5.9–+ 16.1%, respectively) and NPVs (range + 3.7–+ 13.4% and + 2.7–+ 4.2%, respectively) than Reader 1 and Reader 2 without AI. Good interobserver consistency was found between Reader 3 and Reader 4 in interpreting ≥ 50% stenosis (Kappa value = 0.75 and 0.80 at the patient and vessel levels, respectively). Only Reader 1 and Reader 2 showed poor interobserver consistency (Kappa value = 0.25 and 0.37). Reader 5 and Reader 6 showed moderate agreement (Kappa value = 0.55 and 0.61).

**Conclusions:**

Our study showed that using AI could effectively increase the sensitivity of inexperienced readers and significantly improve the consistency of coronary stenosis diagnosis via CCTA.

*Trial registration* Clinical trial registration number: ChiCTR1900021867. Name of registry: Diagnostic performance of artificial intelligence-assisted coronary computed tomography angiography for the assessment of coronary atherosclerotic stenosis.

**Supplementary Information:**

The online version contains supplementary material available at 10.1186/s12880-022-00756-y.

## Background

Many studies have demonstrated the high accuracy of coronary computed tomography angiography (CCTA) compared to invasive coronary angiography (ICA) in detecting coronary stenosis, particularly due to the high sensitivity and negative predictive value (NPV) of CCTA in coronary artery disease diagnosis. However, in previous studies, most of the results were interpreted by cardiovascular experts [1–6]. Reader diagnostic experience significantly impacts the identification of the degree of coronary artery stenosis, as well as the interobserver interpretation variability, with less experienced readers tending to miss lesions, resulting in relatively low diagnostic sensitivity [7, 8]. Less experienced readers also tend to overestimate lesions due to the calcium-blooming effects of hard plaques. Therefore, for radiologists with less cardiovascular experience, the development of an automated system to aid in diagnosis is attractive and promising.

Automated artificial intelligence (AI) algorithms have been applied in the diagnosis of a wide range of disease states [9–14]; for example, the use of AI in the detection and diagnosis of breast cancer and colon polyps has improved reader performance, especially for inexperienced or novice readers [10, 15, 16]. However, AI algorithms are still rarely applied in coronary artery disease [13, 17, 18]. Our study sought to assess the influence of AI based on deep learning on the diagnostic performance and consistency of inexperienced cardiovascular radiologists.

## Methods

### Study population

**Fig. 1:**
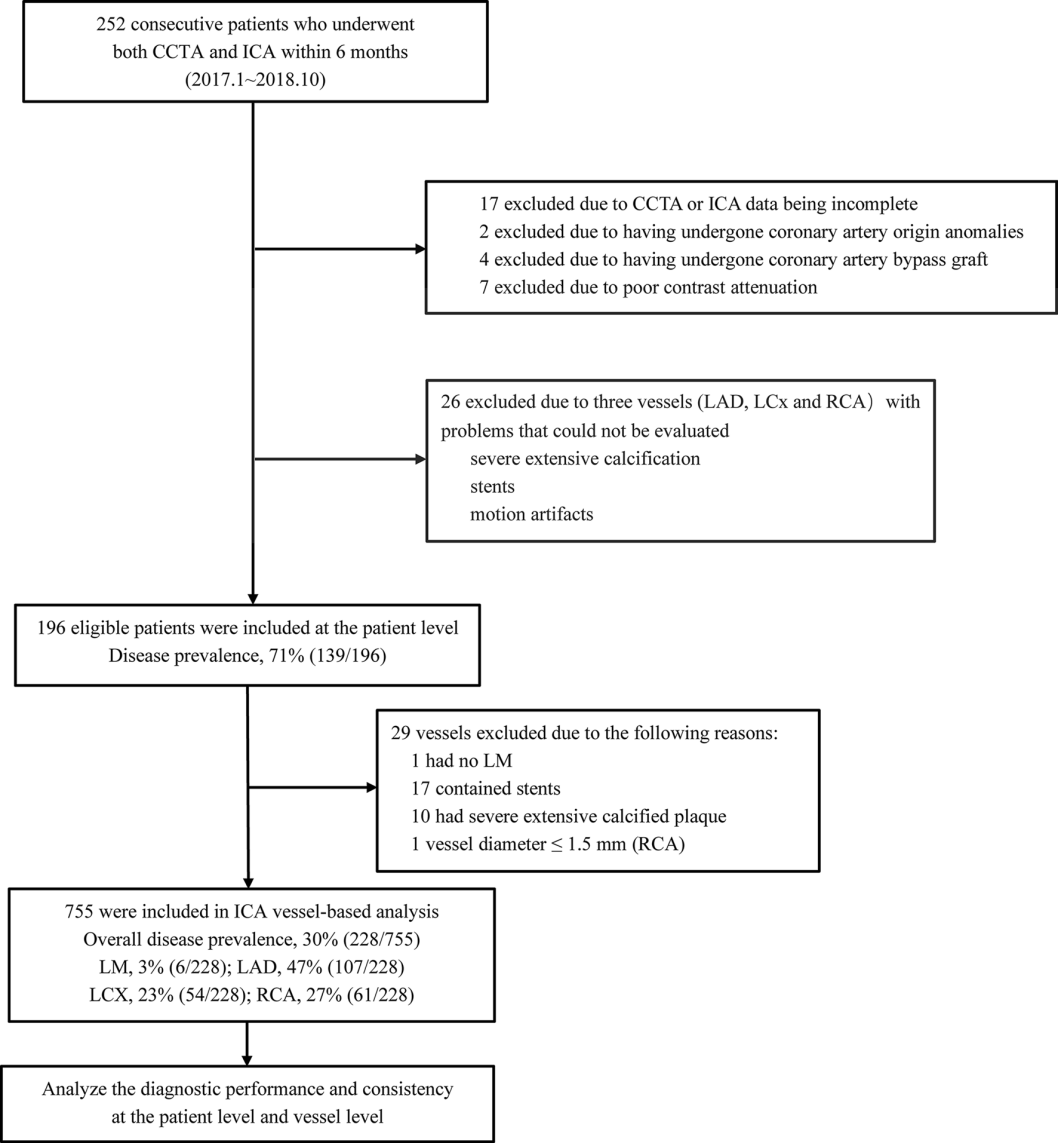
Flow diagram of the patient selection process. *CCTA* coronary computed tomography angiography, *ICA* invasive coronary angiography

This study was a single-centre retrospective study approved by the Ethics Committee of Beijing Friendship Hospital, Capital Medical University (Central Office for Research Ethics Committee Reference 2020-P2-010-02). Between January 2017 and October 2018, 252 consecutive patients (aged over 18 years) with suspected or known coronary heart disease (CHD) underwent both CCTA and ICA examinations within six months. Patients with iodine contrast agent allergy, atrial fibrillation, renal failure or pregnancy were excluded from this study. Of the initially included patients, seventeen had incomplete CCTA or ICA data, 6 had abnormal coronary origins or had undergone bypass surgery, 7 had poor image quality, and 26 had three vessel lesions (left anterior descending (LAD) artery, left circumflex (LCx) artery and right coronary artery (RCA)) that could not be simultaneously evaluated. These patients were excluded due to severely extensive calcification (the standard of severe calcification was cross-sectional arc calcium > 180°) [19], stents and motion artefacts. Finally, 196 patients were enrolled (Fig. 1). ICA is the gold standard of diagnosis and was jointly interpreted for ≥ 50% stenosis by an expert panel of three cardiovascular experts with at least 10 years of experience in both ICA and CCTA.

### CCTA image acquisition

A 256-section CT (GE Healthcare, Waukesha, Wisconsin, US), a 64-section CT (GE Healthcare, Waukesha, Wisconsin, US) and a 128-row multidetector CT (Philips Medical Systems, Eindhoven, The Netherlands) were used to capture patient image data. Prospective electrocardiographic gating was employed. IoproMide (IoproMide, Ultravist 370; Bayer Healthcare LLC, Whippany, New Jersey) or Iohexol (Omnipaque 350, GE Healthcare, Princeton, NJ) was injected at 5–6 ml/s into the antecubital vein. All scanners had a layer thickness and spacing of 0.625 mm.

### CCTA analysis

All 196 CCTA patient datasets were reconstructed at a workstation (GE Advantage Workstation 4.6 or 4.7, GE Healthcare, Waukesha, Wisconsin) to transform the data into multiplanar reformation (MPR) and curved MPR (cMPR) images based on the original axial image. These images were then transferred to a picture archiving and communication system (PACS). Patients were defined as positive for significant coronary artery disease when ≥ 50% stenosis was observed. Six readers interpreted stenosis via CCTA for all patients. The readers had different levels of experience. Four readers (Readers 1 to 4) were general radiologists with less experience in cardiovascular imaging who had interpreted less than 50 cases of coronary artery stenosis via CCTA and had not been mentored [20]. Readers 5 and 6 were cardiovascular radiologists with at least 5 years of CCTA experience, corresponding to level II competency (independent practitioners, IP) [21]. Inexperienced Readers 1 and 2 and experienced Readers 5 and 6 evaluated all patient data on the same PACS without the AI system. Inexperienced Readers 3 and 4 evaluated the same patient data on the AI workstation and received AI assistance in coronary stenosis diagnosis. This study did not require the same readers to interpret the 196 CCTA datasets both with and without AI because CCTA interpretation experience is related to the number of cases evaluated [7, 20, 22]. Interpreting 196 CCTA datasets would have a significant impact on a reader’s experience; therefore, we selected four readers with similar experience levels rather than requiring the same readers to interpret the data twice. Thus, reader recall bias was effectively avoided. The AI system could independently perform automatic reconstruction and intelligently diagnose coronary artery stenosis. All readers analysed the four primary coronary arteries, the left main (LM) artery, LAD, LCx and RCA, and recorded the presence of both vessels and ≥ 50% stenosis. Vessels with severely extensive calcified plaque, stents or a diameter of ≤ 1.5 mm were excluded from this study.

### AI system

#### Data acquisition

The AI system used was “CoronaryDoc clinical decision Support Platform V1.0” from Shukun (Beijing) Technology Co., Ltd. [23]. All CCTA data were transferred from a GE Advantage Workstation 4.6 or 4.7 to the AI workstation, and then the AI system extracted the centerline [24] and automatically reconstructed MPR and cMPR images based on the original axial image.

#### Coronary artery segmentation and naming

Coronary arteries were divided into 18 segments according to the Society of Cardiovascular Computed Tomography (SCCT) criteria [25]. An improved 3-dimensional (3D) U-Net coronary tree segmentation architecture was used. The AI system used an automatic identification algorithm to achieve coronary artery segmentation and naming [24].

#### Automatic reconstruction and intelligent diagnosis of coronary artery stenosis

The system was based on coronary tree segmentation, MPR, straightened rendering (SR), cMPR, maximum intensity projection (MIP) and volume rendering (VR) images, and it automatically reconstructed the data. Stenosis along the long axis of a vessel was calculated based on the radius of the lumen at the plaque location and the upstream and downstream blood vessel radii (details are provided in the Additional file 1: Appendices).

### Statistical analysis

All data were analysed using SPSS (version 26.0, IBM, Washington, USA) and MedCalc (version 19.0.4, bvba, Ostend, Belgium) software. Assuming a 60% prevalence of coronary artery disease at the patient level at our single centre, the area under the receiver operating characteristic curve (AUC) of patients without AI and with AI was 68% and 77%, respectively. The sample size was estimated to be 143 patients, with 86 disease cases and 57 disease-free cases. Categorical variables are shown as percentages, and continuous variables are shown as the means and ranges. We used sensitivity, specificity, the positive predictive value (PPV), the NPV, and the AUC to describe diagnostic performance and accuracy. ICA was used as the gold standard to assess the results of ≥ 50% coronary artery stenosis detection by the 6 readers at the patient and vessel levels. In patient-level analysis, a patient with ≥ 50% stenosis in any vessel or segment was considered a positive case. In vessel-level analysis, any vessel with stenosis ≥ 50% was considered a positive case. AUC comparisons among the 6 readers were performed by the method of DeLong et al. [26]. We used Cohen's kappa coefficient to evaluate the interobserver consistency in detecting ≥ 50% stenosis lesions among the inexperienced readers without AI assistance, the inexperienced readers with AI assistance and the two cardiovascular radiologists. Vessels not evaluated by the six readers or the AI system were not statistically analysed in this study. A P value < 0.05 was considered statistically significant.

## Results

### Patients

**Fig. 2:**
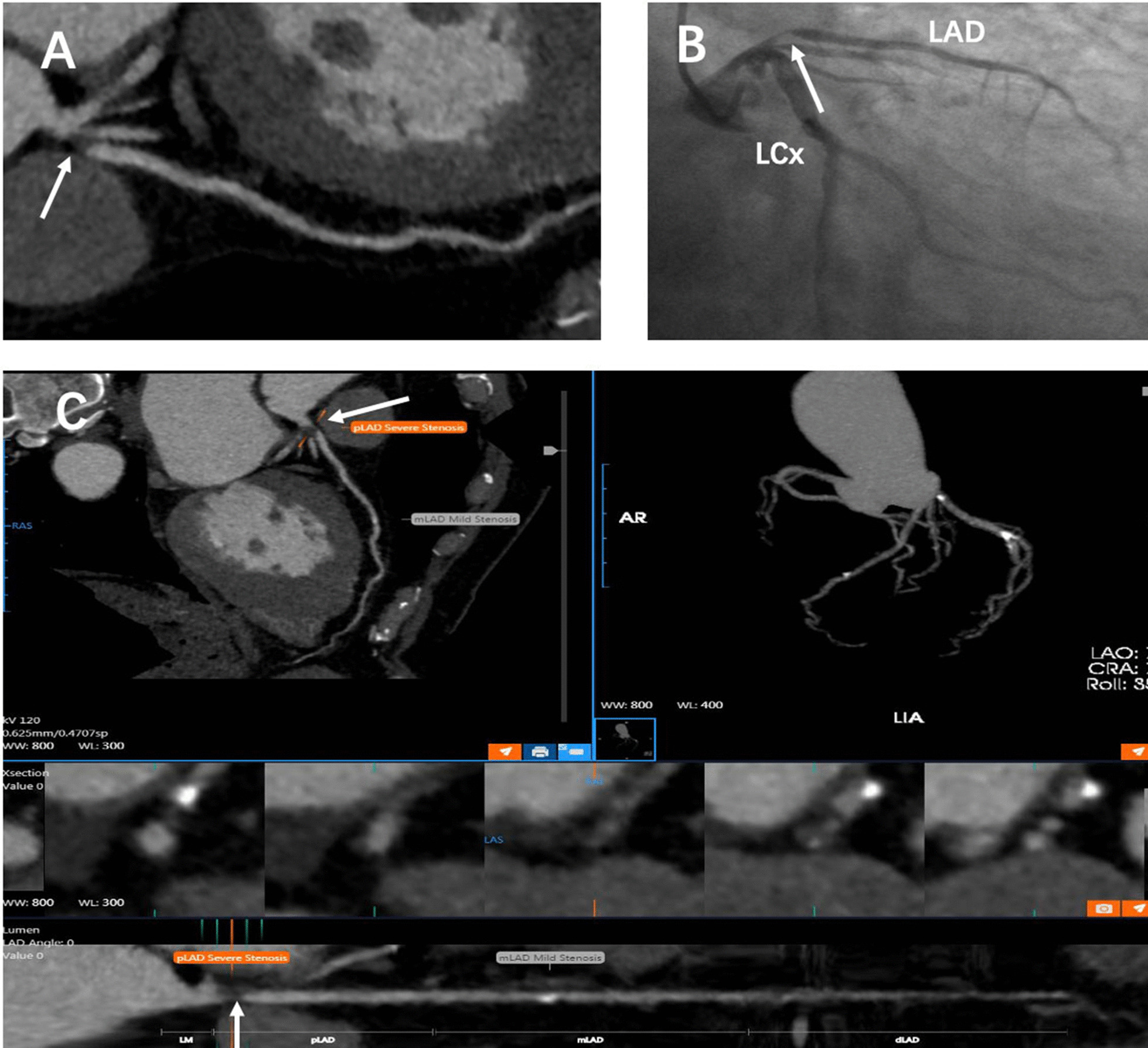
Single-centre retrospective case of a noncalcified plaque lesion in the proximal LAD that caused severe stenosis (white arrow). **a** CPR image reconstructed manually, **b** ICA image of the left coronary artery, **c** CPR, VRT and straightened images automatically reconstructed by the AI system. *ICA* invasive coronary angiography, *LAD* left anterior descending, *LCx* left circumflex

The mean age of the patients was 63.9 ± 8.8 years, and the mean heart rate was 65.2 ± 12.5 beats/min. The clinical information of the patients is shown in Table 1. At the vessel level, seventeen vessels contained stents, ten had severely extensive calcified plaque, one had a vessel diameter ≤ 1.5 mm (RCA), and one had no LM. Finally, 755 vessels were included in the analysis. ICA showed 228 vessel stenoses ≥ 50% in 139 patients (71%), of which six were in the LM, 107 were in the LAD, 54 were in the LCx, and 61 were in the RCA (Fig. 1 and Fig. 2).

**Table 1:** Clinical features of the 196 patients

Parameter	Men	Women
Total no. of patients	124 (63.3%)	72 (36.7%)
Age (y)	61.72 ± 8.50	67.63 ± 8.22
Median body mass index (kg/m^2^)	25.58 ± 3.13	25.93 ± 4.23
No. of patients who smoked	93 (75.0%)	7 (9.7%)
No. of patients who drank	59 (47.6%)	1 (1.4%)
No. of patients with hypertension	89 (72.4%)	54 (75.0%)
No. of patients with hyperlipidaemia	67 (59.3%)	33 (50.8%)
No. of patients with diabetes	52 (43.3%)	27 (37.5%)
No. of patients with known CHD	11 (8.9%)	10 (13.9%)
Heart rate of patients	63.90 ± 11.59	65.18 ± 12.52

No. = number. Age and heart rate are shown as the mean ± standard deviation or the median and range. CHD = coronary heart disease. Hypertension data were available for 195 patients (123 men and 72 women), hyperlipidaemia data were available for 178 patients (113 men and 65 women), and diabetes data were available for 192 patients (120 men and 72 women)

### Patient-level analysis of the six readers

**Fig. 3:**
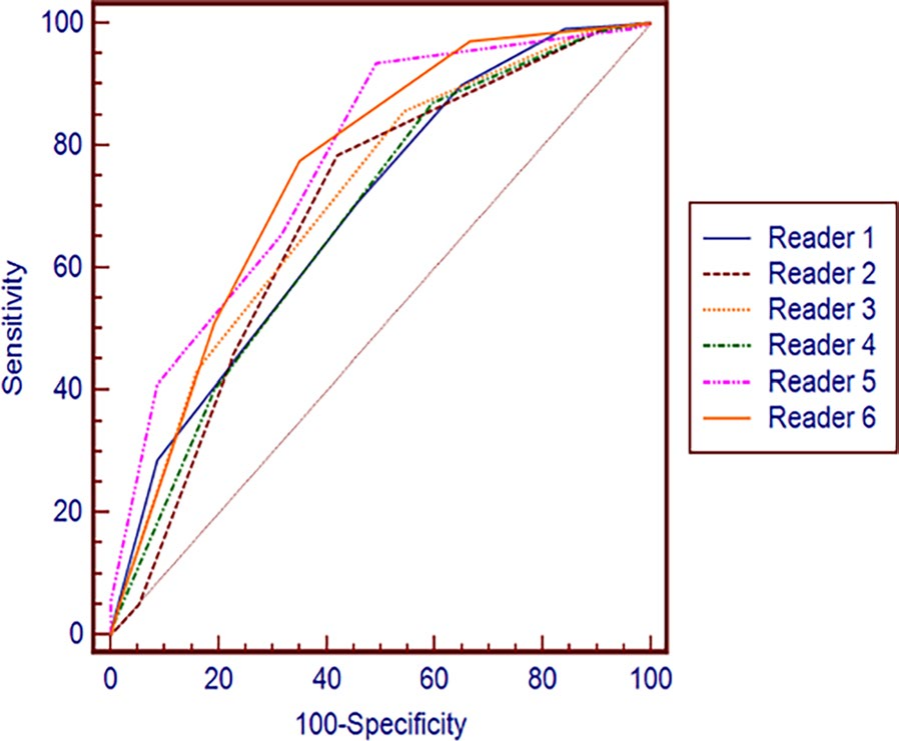
AUC comparison of the six readers at the patient level

Table 2 shows the detailed reader performance and accuracy in identifying ≥ 50% stenosis at the patient level. At the patient level, Readers 3 (85.6%) and 4 (87.1%), who were aided by the AI system, had higher sensitivity than Readers 1 (70.5%) and 2 (78.4%), who did not use the AI system (range + 7.2–+ 16.6%). A statistically significant difference in sensitivity was found between Reader 1 and inexperienced readers with AI assistance (P = 0.001 and P < 0.001). Reader 2 demonstrated no difference in sensitivity (P = 0.164 and P = 0.81), a higher NPV (range + 3.7–+ 13.4%), lower specificity (range − 17.5 to − 8.8%), and no significant difference in PPV. Overall, the AUCs of the four inexperienced readers did not significantly differ (all P values > 0.05). The AUCs, as a measure of diagnostic accuracy, ranged from 0.68 to 0.71 for the inexperienced readers and were lower than those of the experienced readers (that of Reader 5 was 0.77, and that of Reader 6 was 0.76). Of the six readers’ AUCs, only those of Reader 4 and Reader 5 were significantly different (P = 0.02), while the other readers showed no significant differences (Fig. 3).

**Table 2:** Diagnostic performance of the six readers and the AI system alone for the same set of patients

Reader	Sensitivity (%)	Specificity (%)	PPV (%)	NPV (%)	Accuracy (%)
AI	93.5 (88.1, 97.0)	57.9 (44.1, 70.9)	84.4	78.6	80.0 (73.1, 84.9)
Reader 1	70.5 (62.2, 77.9)	54.4 (40.7, 67.6)	79.0	43.1	69.1 (62.1, 75.4)
Reader 2	78.4 (70.6, 84.9)	57. 9 (44.1, 70.9)	82.0	52.4	69.0 (62.0, 75.4)
Reader 3	85.6 (78.7, 91.0)	45.6 (32.4, 59.3)	79.3	56.5	71.2 (64.3, 77.5)
Reader 4	87.1 (80.3, 92.1)	40.4 (27.6, 54.2)	78.1	56.1	67.8 (60.8, 74.3)
Reader 5	65.5 (56.9, 73.3)	68.4 (54.8, 80.1)	83.5	44.8	77.3 (70.8, 83.0)
Reader 6	77.7 (69.9, 84.3)	64.9 (51.1, 77.1)	84.4	54.4	75.6 (69.0, 81.5)

Data in parentheses are 95% confidence intervals

*AI* artificial intelligence, *PPV* positive predictive value, *NPV* negative predictive value

### Vessel-level analysis of the six readers

**Fig. 4:**
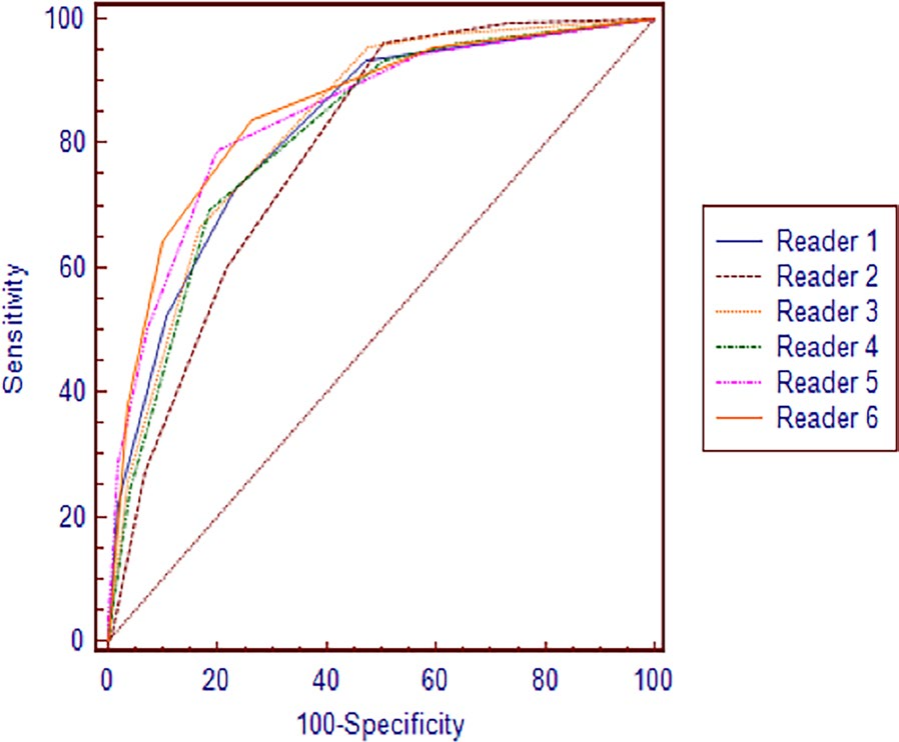
AUC comparison of the six readers at the vessel level

Table 3 shows the detailed reader performance and accuracy in identifying ≥ 50% stenosis at the vessel level. At the vessel level, Readers 3 (67.1%) and 4 (69.3%), who were aided by the AI system, had a higher sensitivity than Readers 1 (53.2%) and 2 (61.2%), who did not have AI assistance (range + 5.9–+ 16.1%). A statistically significant difference in sensitivity was found between Reader 1 and the inexperienced readers with AI assistance (P = 0.001 and P < 0.001), while Reader 2 demonstrated no difference (P = 0.208 and P = 0.76) and a slightly higher NPV (range + 2.7–+ 4.2%). Reader 1 (89.2%) had higher specificity than Readers 3 (83.2%) and 4 (81.3%), while Reader 2 (78.3%) had slightly lower specificity than Readers 3 and 4. Overall, the AUCs of the four inexperienced readers did not significantly differ (all P value > 0.05). The AUC, as a measure of diagnostic accuracy, ranged from 0.80 to 0.83 for the inexperienced readers and was lower for the experienced readers (those of Readers 5 and 6 were both 0.85). Of the six readers’ AUCs, only the AUC of Reader 2 was significantly different from those of the experienced readers (P = 0.009 and P = 0.002), while Readers 4 and 6 showed slightly statistically significant differences (P = 0.045). The AUCs of other readers did not significantly differ (Fig. 4).

**Table 3:** Diagnostic performance of the six readers and the AI system alone for the same set of vessels

Reader	Sensitivity (%)	Specificity (%)	PPV (%)	NPV (%)	Accuracy (%)
AI	78.1 (72.1, 83.3)	82.5 (79.0, 85.7)	65.9	89.7	84.5 (81.7, 87.0)
Reader 1	53.2 (46.4, 59.9)	89.2 (86.2, 91.7)	67.8	81.7	82.5 (79.6, 85.2)
Reader 2	61.2 (54.6, 67.6)	78.3 (74.6, 81.8)	54.9	82.7	79.5 (76.4, 82.3)
Reader 3	67.1 (60.6, 73.2)	83.2 (79.8, 86.3)	63.5	85.4	83.3 (80.4, 85.9)
Reader 4	69.3 (62.9, 75.2)	81.3 (77.7, 84.5)	61.7	85.9	81.3 (78.4, 84.1)
Reader 5	50.9 (44.2, 57.6)	93.1 (90.5, 95.1)	76.2	81.3	84.7 (81.9, 87.2)
Reader 6	64.2 (57.5, 70.4)	89.8 (86.9, 92.3)	73.2	85.2	85.0 (82.2, 87.5)

Data in parentheses are 95% confidence intervals

*AI* artificial intelligence, *PPV* positive predictive value, *NPV* negative predictive value

### Diagnostic performance of the AI system

The CCTA data of all 196 patients were successfully uploaded and automatically reconstructed. To identify ≥ 50% stenosis, the AI system could output the degree of stenosis in each present vessel and segment. At the patient level, the AI system alone had 93.5% sensitivity, 57.9% specificity, 84.4% PPV, 78.6% NPV and 80.0% accuracy (Table 2). At the vessel level, the AI system alone had 78.1% sensitivity, 82.5% specificity, 65.9% PPV, 89.7% NPV and 84.5% accuracy (Table 3). Compared to ICA, AI indicated 9/139 (6.5%) false-positive and 24/57 (42.1%) false-negative patients at the patient level and 50/228 (21.9%) false-positive and 92/526 (17.5%) false-negative vessels at the vessel level. The AI system missed one lesion (LCx) at the vessel level, while no lesions were missed at the patient level. Overall, the diagnostic accuracy of the AI system was slightly higher than that of the experienced readers (IP) at the patient level and close to that of the experienced readers at the vessel level.

### Diagnostic consistency of the six readers

Good interobserver consistency was found between the two inexperienced readers aided by the AI system (Readers 3 and 4) in interpreting lesions with more than 50% stenosis, with Kappa values of 0.75 (95% CI 0.64, 0.86) and 0.80 (95% CI 0.75, 0.84) at the patient and vessel levels, respectively. However, the two inexperienced readers without AI assistance (Readers 1 and 2) had very poor interobserver consistency, with kappa values of 0.25 (95% CI 0.11, 0.38) and 0.37 (95% CI 0.30, 0.44) at the patient and vessel levels, respectively. In interpreting lesions with more than 50% stenosis, the experienced cardiovascular radiologists without AI assistance (Readers 5 and 6) showed moderate agreement, with kappa values of 0.55 (95% CI 0.43, 0.66) and 0.61 (95% CI 0.54, 0.68) at the patient and vessel levels, respectively.

## Discussion

Automated computerized detection and diagnostic systems have been introduced as auxiliary tools for radiologists in diverse diagnostic processes [9–14]. Coronary artery disease is one of the leading causes of life-threatening health problems in developing countries [27, 28], but AI is still rarely used for this disease diagnosis [18]. At present, several studies have compared the diagnostic performance of AI with that of ICA or experts [17, 29]. The preliminary conclusion is that AI has great value and promise for application and may be used as a diagnostic aid by inexperienced radiologists. The influence of AI on the diagnostic performance and consistency of inexperienced readers has relatively rarely been studied for the diagnosis of coronary artery disease with ≥ 50% stenosis.

Our results indicated that the use of AI as a diagnostic aid might have a positive effect on inexperienced radiologists in diagnosing coronary stenosis on CCTA. The most obvious effect was that inexperienced readers with AI assistance performed better than those without AI assistance at both the patient and vessel levels, and this difference was statistically significant. Additionally, the NPV of the former was also higher. Moreover, the consistency of inexperienced readers was significantly higher. When inexperienced readers used the AI system as an auxiliary reader, their sensitivity reached or surpassed that of more experienced readers (IP). In our analysis, based on the mildly improved diagnostic accuracy observed when using the AI system, the sensitivity and NPV were significantly higher, without a significant decrease in specificity or PPV. These findings were similar to those of previous studies evaluating the effect of AI in diagnosing coronary artery disease and other diseases [10, 13, 16]. Moreover, the AI training of less experienced radiologists was also dominated by an increase in sensitivity, which is in line with the results of previous studies [7, 30]. Increased sensitivity of less experienced readers could improve radiologists’ abilities to detect obstructed coronary arteries and reduce missed disease diagnoses. The reason for the increased sensitivity of inexperienced readers may be their tendency to rely the AI system when unsure of the presence of obstructive disease. Consequently, the AI system, with high sensitivity (93.5% at the patient level) and low specificity (57.9% at the patient level), also reflected the performance of inexperienced readers. Importantly, AI assistance clearly increased the consistency of the inexperienced readers (the kappa value increased from 0.25 to 0.75 at the patient level).

Experienced readers (IP) had higher diagnostic accuracy than inexperienced readers, suggesting that the reader’s experience influenced their diagnostic performance and that specific cardiovascular training is important. In clinical work, specialty training, although important, is time-consuming and demanding, especially for inexperienced practitioners. With the increasing use of deep learning algorithms, at present, AI has achieved higher sensitivity but lower specificity at the patient level. Therefore, the AI system had similar limitations to those of human readers in that it could not accurately measure the severity of lesions and overestimated them compared to ICA or quantitative coronary angiography (QCA) [31]. The algorithm is still undergoing optimization. Considering these factors, AI assistance still holds great potential for improving disease detection and excluding stenosis at the patient level for less experienced readers or novices, and it may provide an appropriate training alternative.

Our study had several limitations. First, we used ICA as the reference standard, and enrolled patients who underwent ICA were likely to have severe stenosis, which might have led to a high disease prevalence (71% at the patient level); thus, this study is subject to the same selection bias shown in previous comparative studies [13]. Second, we chose 50% coronary stenosis as the cut-off value based on previous research, but this analysis was different from actual clinical practice [13, 32]. Third, due to the relatively large number of patients enrolled and considering that case interpretation may have impacted inexperienced readers, diagnostic performance was not compared among the same readers with and without AI assistance. Instead, we selected different readers with similar experience levels, which might have impacted the obtained results; however, the overall trend was consistent with those of previous studies using the same readers for both tasks. Therefore, the study design effectively avoided methodological reader recall bias.

In conclusion, as a supplement, the AI system could effectively increase the diagnostic sensitivity of less experienced readers and significantly improve their consistency.

## Supplementary Information

**Additional file 1.** Deep-learning model (AI system).

## Data Availability

The datasets used and analysed during the current study are available from the corresponding author upon reasonable request. All data generated or analysed during this study are included in this published article [and its supplementary information files].

## Abbreviations

CCTA: Coronary computed tomography angiography
ICA: Invasive coronary angiography
AI: Artificial intelligence
CHD: Coronary heart disease
PACS: Picture archiving and communication system
IP: Independent practitioners
SCCT: Society of Cardiovascular Computed Tomography
