# Supplementary material for: Artificial intelligence stenosis diagnosis in coronary CTA: effect on the performance and consistency of readers with less cardiovascular experience
Source: BMC Med Imaging. 2022 Feb 17;22:28. doi: 10.1186/s12880-022-00756-y (PMC8851787; doi:10.1186/s12880-022-00756-y)
Supplement: Supplementary file 1 — Additional file 1. Deep-learning model (AI system). [file 12880_2022_756_MOESM1_ESM.docx]

**Deep-learning model (AI system)**

An imaging reconstruction system (CoronaryDoc, ShuKun Technology, Beijing) based on an optimized anatomy prior-knowledge-based deep-learning model, which was divided into two parts, coronary tree segmentation and stenosis detection, was used for automated postprocessing and assessment of coronary stenosis. In brief, more than 10,000 coronary computed tomography angiography (CCTA) scan were used to training (70%), tuning (20%) and validating (10%) the deep-learning algorithm.

The coronary tree segmentation architecture used was an improved 3D U-Net [1]. The improved 3-dimensional (3D) U-Net architecture combined with a Bottle-Neck design was used for coronary artery and aorta segmentation. Then, a connected growth prediction model (CGPM) was developed to solve the problem of vascular segmentation fracture. Finally, full coronary tree segmentation was obtained. The original 3D U-Net architecture has four layers each for the encoder and decoder; to improve the architecture of 3D U-Net, a bottle-neck design with 1$\times$1, 3$\times$3 and 1$\times$1 convolutions was added between the middle two layers. The CGPM model used a crop size of 64$\times$64$\times$64 for vascular segmentation fracture sites and applied 3D U-Net architecture for repairing the fractures.

Based on coronary tree segmentation, multiple planar reformation (MPR), straightened rendering (SR), curved planar reformation (CPR), maximum intensity projection (MIP) and volume rendering (VR) images were reconstructed. A fully automatic identification algorithm for coronary arteries based on the Society of Cardiovascular Computed Tomography (SCCT) standard was applied to label the coronary arteries [2]. Atherosclerotic plaque can be classified into calcified plaque, noncalcified plaque, and mixed plaque according to its composition [3]. Another two U-Net models were trained to detect calcified plaques on CPR images and noncalcified and mixed plaques on SR images. Stenosis along the long axis of the vessel was calculated based on the radius of the lumen where the plaque was located and the radius of the upstream and downstream blood vessels:

*N* _= 2*_*_RS/_*_(_*_R_*_1+_*_R_*_2)_

where *R_S_* is the radius of the lumen where the plaque is located and *R*_1_ and *R*_2_ are the radii of the upstream and downstream blood vessels, respectively.

The model training process included both coronary tree segmentation and stenosis detection. Dice loss, calculated by subtracting the mean Dice similarity score from 1, was used to train the network [4]. The 3D U-net architecture was trained using the SGD optimizer with a momentum of 0.95, a peak learning rate of 0.1 for randomly initialized weights, a weight decay of 0.0001, and an initial learning rate of 0.01 that shrank by 0.99995 after each training step of 200,000 iterations. The network with the lowest Dice loss on the validation set was selected and evaluated.

References:

1. Çiçek, Özgün et al. “3D U-Net: Learning Dense Volumetric Segmentation from Sparse Annotation.” *MICCAI* (2016).
2. Zhang C J, Xia D, Zheng C, et al. Automatic Identification of Coronary Arteries in Coronary Computed Tomographic Angiography[J]. IEEE Access, 2020, 8: 65566-65572.
3. G. L. Raff et al., "SCCT guidelines for the interpretation and reporting of coronary computed tomographic angiography", J. Cardiovascular Comput. Tomogr., vol. 3, no. 2, pp. 122-136, 2009.
4. Sudre C H , Li W , Vercauteren T , et al. Generalised Dice Overlap as a Deep Learning Loss Function for Highly Unbalanced Segmentations[C]// International Workshop on Deep Learning in Medical Image Analysis International Workshop on Multimodal Learning for Clinical Decision Support. 2017.
